# Une cytolyse hépatique révélant un ganglioneurome surrénalien

**DOI:** 10.11604/pamj.2014.17.224.3744

**Published:** 2014-03-21

**Authors:** Hicham Baïzri, Youssef Zoubeir, Sanae Elhadri, Jihad Elanzaoui, Zakaria Chahbi, Said Kaddouri, Hassan Qacif, Driss Touiti, Mohammad Zyani

**Affiliations:** 1 Service d'Endocrinologie Diabétologie et Maladies Métaboliques, Hôpital Militaire Avicenne, Marrakech, Maroc; 2 Laboratoire d'Anatomie Pathologique, Hôpital Militaire Avicenne, Marrakech, Maroc; 3 Service d'Urologie, Hôpital Militaire Avicenne, Marrakech, Maroc; 4 Service de Médecine Interne, Hôpital Militaire Avicenne, Marrakech, Maroc

**Keywords:** Ganglioneurome, tumeurs rétropéritonéales, TDM, IRM, ganglioneuroma, retroperitoneal tumors, CT, MRI

## Abstract

Le ganglioneurome est une tumeur nerveuse bénigne rare, d'origine neuroectodermique et de localisation rétropéritonéale fréquente. Nous rapportons l'observation d'un patient de 55 ans dont la tumeur est révélée fortuitement sur une échographie abdominale demandée dans le cadre d'une cytolyse hépatique secondaire à une hépatite virale C. Le patient est opéré après la réalisation d'un scanner abdominal et d'un bilan hormonal. L'examen anatomopathologique de la pièce opératoire est en faveur d'un ganglioneurome. Devant une volumineuse masse rétropéritonéale avec état général conservé, on doit envisager le diagnostic de ganglioneurome car l'exérèse chirurgicale complète permet une guérison sans récidive. Préalablement, l'ensemble des autres diagnostics différentiels doit être éliminé.

## Introduction

Le ganglioneurome est une tumeur nerveuse bénigne de l'enfant et de l'adulte jeune. C'est une tumeur rare, localisée dans la glande surrénale (20%), le long de la chaîne sympathique et particulièrement au niveau du médiastin postérieur (40%) et du rétropéritoine (30%) [1]. Elle appartient au groupe des tumeurs neurogènes, se développant aux dépens des chaînes ganglionnaires sympathiques, groupe qui inclut également les ganglioneuroblastomes et les neuroblastomes [2]. En effet, celles-ci posent un problème de diagnostic clinique et histologique, ainsi qu'un problème thérapeutique à cause des rapports avec les organes de voisinage, et notamment avec les gros vaisseaux (veine cave inférieure, aorte). Nous rapportons l'observation d'un patient âgé de 55 ans pour lequel le diagnostic de ganglioneurome surrénalien droit est révélé fortuitement dans le cadre du bilan d'une cytolyse hépatique.

## Patient et observation

**Figure 1:**
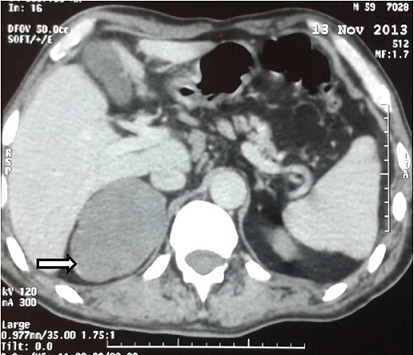
TDM abdominale sans injection du produit de contraste montrant une masse de la loge surrénalienne droite bien limitée et homogène (flèche blanche)

**Figure 2:**
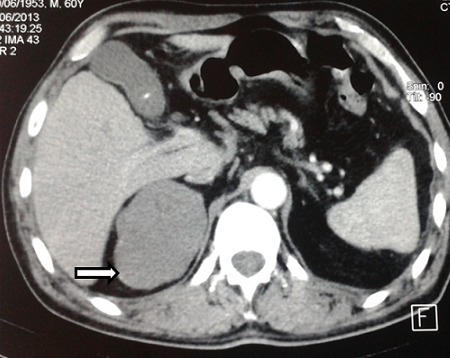
TDM abdominale avec injection du produit de contraste montrant une masse de la loge surrénalienne droite bien limitée et homogène ne prenant pas le produit de contraste (flèche blanche)

Un patient âgé de 55 ans, militaire de fonction, est adressé en consultation de médecine interne pour visite de réengagement. Dans ses antécédents personnels nous retrouvons une blessure de guerre superficielle avec persistance d’éclats métalliques au niveau thoracique. L'interrogatoire ne révèle aucune plainte somatique. L'examen clinique trouve une TA normale sans hypotension orthostatique et il n'y a pas de signes cliniques en faveur d'un syndrome d'hyperfonctionnement cortico ou médullosurrénalien, ni signes d'insuffisance surrénalienne. Les examens biologiques révèlent une hyperglycémie à 8,54 mmol/l, une légère cytolyse hépatique avec des ASAT à 66 puis à 88 UI/l (VN <50) et des ALAT à 72 puis à 73 UI/l (VN < 65), la sérologie de l'hépatite C (HVC) est positive avec une charge virale à 6,31 Log (2,04 E + 6 UI/ml), un génotype 1b, et stade F4-A2 au Fibro et Acti tests. Sur le plan radiologique, la radiographie thoracique systématique est sans anomalies notables alors que l'échographie, demandée dans le cadre de la cytolyse et de l'HVC, révèle la présence d'une énorme masse hypoéchogène de contours polylobés (71x70 mm) au niveau de la loge surrénalienne droite. Le complément scannographique confirme la présence de la masse surrénalienne droite qui est ovalaire bien limitée, mesurant 91x59 mm de densité tissulaire, non rehaussée après injection du produit de contraste, refoulant la veine cave inférieure vers l'avant et abaissant le rein homolatéral avec un plan de clivage respecté avec les organe de voisinage (Figure 1, Figure 2). L'IRM surrénalienne ne peut pas être réalisée du fait de la persistance d'éclats métalliques. Le cortisol de 8h, le cortisol libre urinaire des 24 heures ainsi que les dérivés méthoxylés urinaires des 24 heures sont normaux. Le patient bénéficie d'une exérèse chirurgicale de la tumeur par laparotomie transpéritonéale vue la taille de la tumeur avec à l'examen anatomopathologique un aspect en faveur d'un ganglioneurome.

## Discussion

Le ganglioneurome est une tumeur bénigne rare d'origine neuroectodermique, développée à partir du système nerveux sympathique. Il est composé de cellules ganglionnaires matures et d'un stroma contenant des cellules nerveuses associées à un contingent schwannien, contrairement au neuroblastome et au ganglioneuroblastome qui sont composés de cellules ganglionnaires plus immatures dont le potentiel évolutif est plus important [3]. Le ganglioneurome est rencontré le plus souvent chez l'enfant et l'adulte jeune [4, 5]. Le sexe féminin est plus souvent atteint avec un sexe ratio de 0,75 [3]. Chez l'enfant, le ganglioneurome peut représenter la forme mature d'un neuroblastome. A l'inverse, la transformation d'un ganglioneurome en neuroblastome à l’âge adulte est exceptionnelle [2]. Le ganglioneurome se développe donc le long des chaînes sympathiques, d'où ses localisations cervicale, médiastinale, rétropéritonéale ou pelvienne. La localisation rétropéritonéale est fréquente (32-52%) [3, 6], une fois sur deux extrasurrénalienne. Le ganglioneurome ne représente que 0,7 à 1,6% des tumeurs rétropéritonéales primitives [2] qui, quant à elles, constituent moins de 1% de l'ensemble des tumeurs [2]. Le mode de révélation est souvent fortuit, comme c'est le cas de notre patient, car malgré leur grande taille, les ganglioneuromes rétropéritonéaux sont généralement asymptomatiques. Parfois une douleur abdominale, la palpation d'une masse abdominale, ou la compression des organes de voisinage mènent au diagnostic [5]. En fait, Cronin et al. rapportent un cas d'occlusion intestinale par un ganglioneurome survenant 18 ans après le diagnostic initial [7].

L'aspect du ganglioneurome en imagerie est assez évocateur: tumeur ovoïde bien limitée, aux contours réguliers et au contenu très homogène, peu vascularisée, n'envahissant pas les structures vasculaires de voisinage, située le long des chaînes sympathiques, a- ou pauci-symptomatique chez un sujet jeune ou d’âge moyen. L’échographie est peu spécifique et met souvent en évidence une masse tissulaire, hétérogène, à contours bien définis, de la loge surrénalienne. La tumeur peut venir à proximité des vaisseaux sans les envahir [6]. La tomodensitométrie peut mettre en évidence des calcifications dans 50% des cas. Il n'y a pas de contingent graisseux ni kystique. La prise de contraste est faible. En IRM, le ganglioneurome est en hypo-signal homogène en pondération T1, en hyper ou isosignal en pondération T2 selon la quantité de stroma contenu dans la lésion. Les calcifications sont mal analysables par cette technique. [6]. La prise de contraste après injection de Gadolinium n'est pas spécifique, allant de l'absence de prise de contraste à une faible prise inhomogène ou même parfois à une très forte prise [3]. Le bilan hormonal est dans la grande majorité des cas normal. Dans de rares cas, une sécrétion de catécholamines ou de vasoactive intestinale polypeptide (VIP) a été rapportée, responsable de diarrhée et d'hypertension artérielle [6].

Les diagnostics différentiels sont nombreux face à une lésion rétropéritonéale tissulaire de grande taille se développant entre des structures normales [6], mais la prise en compte des nuances sémiologiques et du contexte de découverte aide à réduire l’éventail à envisager. Ainsi, face à une masse rétropéritonéale solide et homogène chez un patient asymptomatique, les seuls diagnostics plausibles sont ceux de maladie de Castleman unicentrique vasculo-hyaline [6] ou de paragangliome non sécrétant si la tumeur est hypervascularisée, et de ganglioneurome en cas de lésion paucivasculaire, ce qui est le cas ici. Les autres diagnostics doivent être évoqués de principe, et aussitôt éliminés pour des raisons sémiologiques ou épidémiologiques. Parmi les tumeurs bénignes, le tératome et le lipome ont un contingent graisseux, le schwannome a souvent une composante kystique, la tumeur desmoïde est mal limitée, le lymphangiome kystique est liquidien et le séminome rétropéritonéal (tumeur burned-out) s'accompagne d'une altération de l’état général. Les lésions malignes (primitives, secondaires, lymphomateuses) sont multiples, souvent nécrotiques, et envahissent les structures de voisinage. Si les différents éléments sont non informatifs une ponction biopsie sera envisagée. Ce geste doit être pesé car il n'est pas dénudé de risque et on retrouve de rares complications liées à la voie d'abord (pneumothorax, pancréatite aigue, hémorragie, infection ou lésion des organes de voisinages) [8]. Néanmoins, le diagnostic de certitude ne sera porté qu'après une étude histologique de la pièce chirurgicale. En effet, la biopsie préopératoire, bien qu'elle permette de poser le diagnostic, une analyse complète de la pièce d'exérèse reste nécessaire en raison de la possibilité de contingents de neuroblastome mais aussi de phéochromocytome au sein du ganglioneurome [2].

Le traitement reste chirurgical et consiste à l'exérèse tumorale; intervention d'autant plus difficile que la tumeur est de grande taille présentant des rapports intimes avec les structures voisines, notamment les gros vaisseaux (VCI et aorte) [3]. Le traitement devrait être réalisé précocement non seulement pour confirmer la nature de la masse, mais aussi pour prévenir l'augmentation de son volume et la compression des structures adjacentes. La voie d'abord est généralement une laparotomie transpéritonéale, essentiellement pour les grosses masses [6] comme chez notre patient. La voie coelioscopique reste possible et même privilégiée pour les petites masses rétropéritonéales bien définies sans rapport intime avec les gros vaisseaux. L’évolution de ces tumeurs est lente, mais l'augmentation de volume est la règle en l'absence de traitement. Leur pronostic est bon en cas d'exérèse complète. Les complications sont surtout d'ordre mécanique [6]. La récidive locale est exceptionnelle, cependant la possibilité d'une transformation maligne en un ganglioneuroblastome est possible, d'où l'intérêt d'une surveillance prolongée [1].

## Conclusion

Malgré sa rareté et sa bénignité, le ganglioneurome rétropéritonéal mérite d’être connu. Le diagnostic est souvent tardif. L'imagerie, en particulier la TDM et l'IRM confirment le siège rétropéritonéal de la tumeur, ses rapports et prédisent de sa réséquabilité. Son pronostic extrêmement favorable après chirurgie justifie l'exérèse complète. Les récidives locales bien que rares imposent une surveillance périodique.
